# [Corrigendum] Overexpression of lncRNA AFAP1-AS1 promotes cell proliferation and invasion in gastric cancer 

**DOI:** 10.3892/ol.2026.15713

**Published:** 2026-06-18

**Authors:** Zhouxiao Li, Zhili Ding, Dawei Rong, Weiwei Tang, Hongyong Cao

Oncol Lett 18: 3211–3217, 2019; DOI: 10.3892/ol.2019.10640

Subsequently to the publication of the above paper, an interested reader drew to the authors’ attention that, concerning the scratch-wound assay experiments shown in Fig. 5A on p. 3216, the ‘12 h/NC’ and ‘12 h/siRNA1’ data panels contained an overlapping section, such that these data appeared to have been derived from the same original source where the results from two different cell lines were intended to have been portrayed. In addition, the reader noted that the MGC-803 cell line had been used in various of the experiments, which has been reported to be a problematic (contaminated) cell line according to the website Cellosaurus on account of its being a hybrid of HeLa, and probably of a gastric cancer cell from an Asian individual, a poorly differentiated gastric mucinous adenocarcinoma.

The authors were able to re-examine their original data, and realized that the abovementioned data featured in Fig. 5A had inadvertently been selected incorrectly. A revised version of Fig. 5, now showing data for these experiments performed using the AGS cell line instead of the problematic MGC-803 cell line, is shown on the subsequent pages. In addition, considering that Figs. 3 and 4 also portrayed experiments performed exclusively with the MGC-803 cell line, new versions of Figs. 3 and 4 showing alternative experiments with the AGS cell line (namely, AFAP1 expression and cell viability following AGS knockdown, and the effects of AFAP1-AS1 on apoptosis and the cell cycle in AGS cells, for Figs. 3 and 4 respectively) are also shown on the subsequent two pages. The authors confirm that the results obtained from the newly performed experiments were broadly similar to the results shown for these three figures in the published version of this article: In Fig. 3, with the AGS cell line, compared with the NC group, the knockdown efficiency of siRNA1 was ~70%, and for siRNA2 it was ~65%. In Fig. 4A and B, no significant difference was found in the rate of apoptosis between the NC and siRNA1 groups, although the apoptotic rate of the AGS cells was significantly increased in the siRNA2 group (P<0.01); moreover, the proportion of cells in the G0/G1 phase following APAP1-AS1 knockdown was similarly shown to be significantly increased in the siRNA1 and siRNA2 groups compared with that observed in the NC group. Finally, in Fig. 5, the migratory ability of the AGS cell line was significantly inhibited following knockdown of AFAP1-AS1.

The authors regret that errors that were made while compiling the original figures, in addition to their the oversight in using a contaminated cell line for various of the experiments. They are grateful to the editor of *Oncology Letters* for allowing them the opportunity to publish this corrigendum. All the authors agree with the publication of this corrigendum; furthermore, they apologize to the readership for any inconvenience caused.

## Figures and Tables

**Figure 3.**
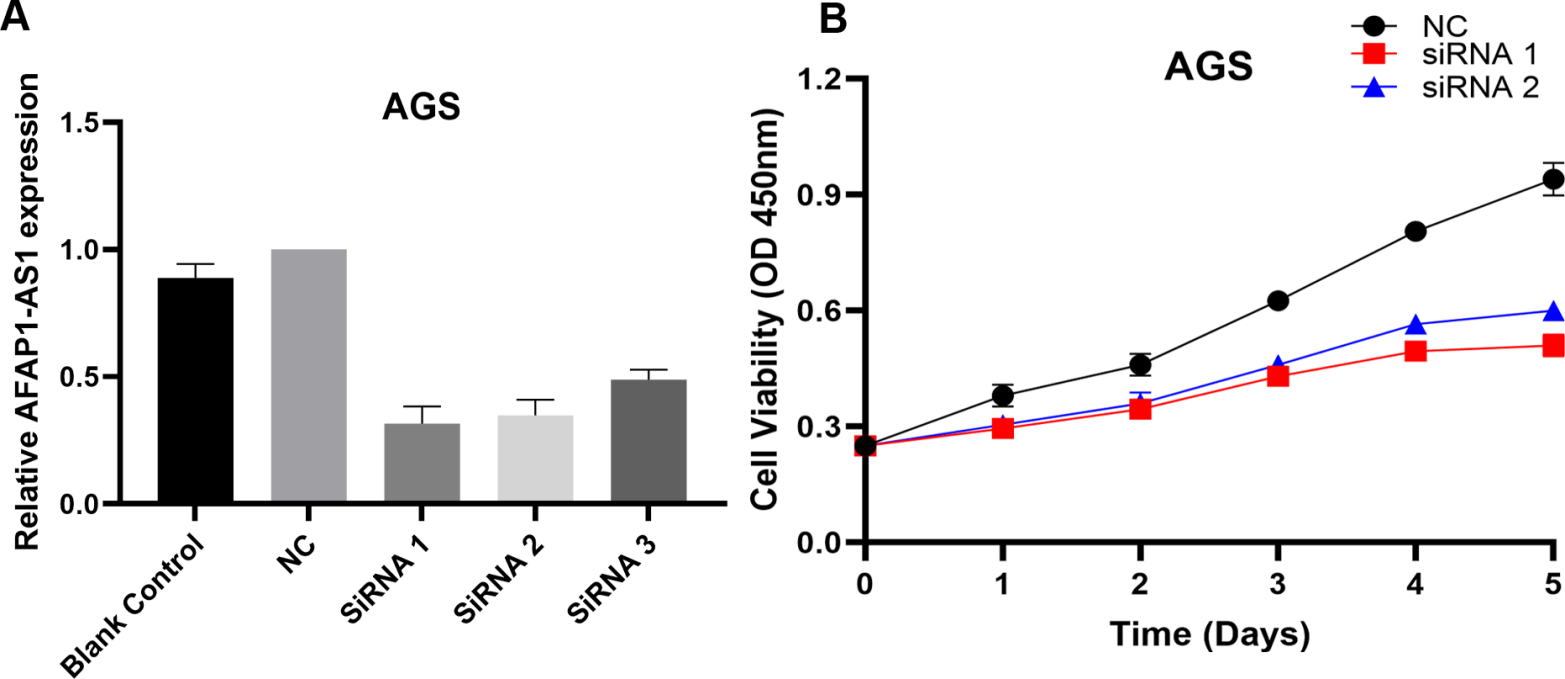
The AFAP1 expression and cell viability following AGS knockdown. (A) Detection of AFAP1-AS1 knockdown efficiency in the gastric cancer cell line AGS. Compared with the NC group, the knockdown efficiency of siRNA1 was ~70% and for siRNA2 was ~65%. (B) MTT assays in the AGS cell line following alteration of the expression of AFAP1-AS1. AFAP1-AS, actin filament-associated protein 1 antisense RNA 1; siRNA, small interfering RNA; NC, negative control; OD, optical density. **P<0.01 vs. control..

**Figure 4.**
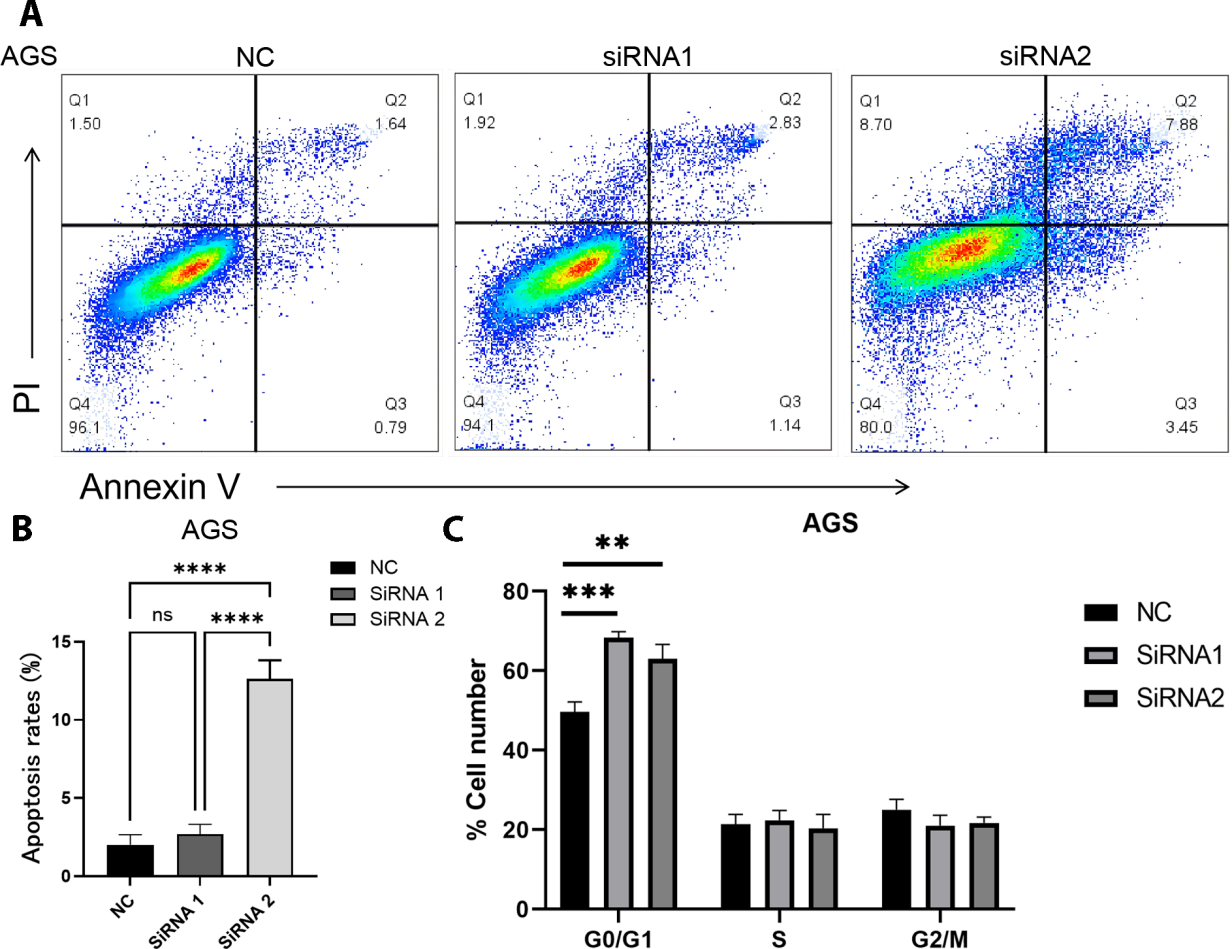
Effects of AFAP1-AS1 on apoptosis and the cell cycle in AGS cells. (A and B) Flow cytometry of apoptosis in AGS cells transfected with siRNA1 and siRNA2. The specific AFAP1-AS1 siRNA2 increased the rate of apoptosis in AGS cells. (A) Representative scatter plot demonstrating levels of apoptosis. (B) Quantification of flow cytometry data. (C) Cell cycle phase distributions were analyzed using flow cytometry. AFAP1-AS, actin filament-associated protein 1 antisense RNA 1; siRNA, small interfering RNA; NC, negative control; FITC, fluorescein isothiocyanate; PI, propidium iodide. *P<0.05 and **P<0.01 vs. negative control.

**Figure 5.**
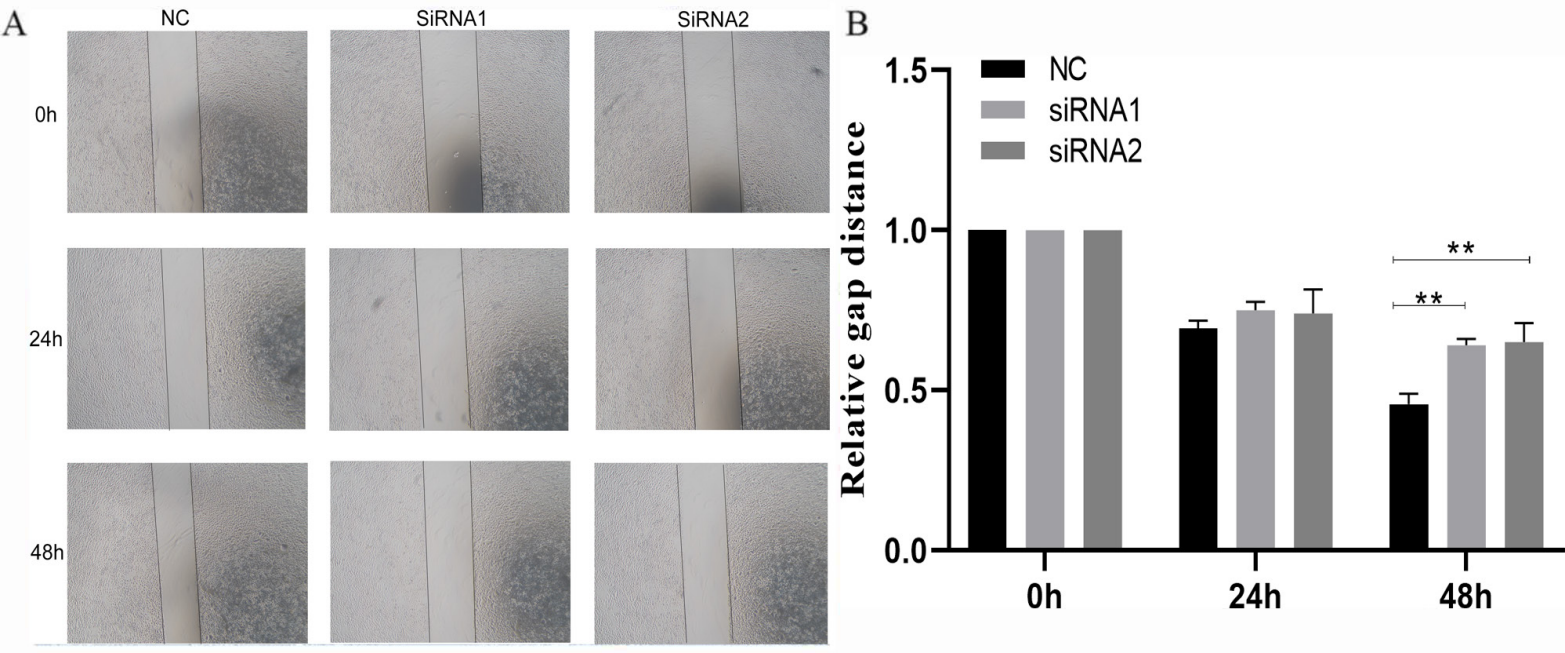
Effects of AFAP1-AS1 on the migratory ability of the gastric cancer AGS cell line. (A) Wound healing assays were conducted to assess the migratory capabilities of AGS cells transfected with si-AFAP1-AS1. (B) The data are summarized as the relative gap distance. AFAP1-AS, actin filament-associated protein 1 antisense RNA 1; siRNA, small interfering RNA; NC, negative control. **P<0.01 vs. NC..

